# Robust PDF Watermarking against Print–Scan Attack

**DOI:** 10.3390/s23177365

**Published:** 2023-08-23

**Authors:** Lei Li, Hong-Jun Zhang, Jia-Le Meng, Zhe-Ming Lu

**Affiliations:** 1 Command and Control Engineering College, Army Engineering University, Nanjing 210007, China; ll605531524@163.com (L.L.); xdrmxxjs@163.com (H.-J.Z.); 2 School of Aeronautics and Astronautics, Zhejiang University, Hangzhou 310027, China; mengjiale@zju.edu.cn

**Keywords:** digital watermarking, PDF protection, leak tracing, robustness, print–scan attack

## Abstract

Portable document format (PDF) files are widely used in file transmission, exchange, and circulation because of their platform independence, small size, good browsing quality, and the ability to place hyperlinks. However, their security issues are also more thorny. It is common to distribute printed PDF files to different groups and individuals after printing. However, most PDF watermarking algorithms currently cannot resist print–scan attacks, making it difficult to apply them in leak tracing of both paper and scanned versions of PDF documents. To tackle this issue, we propose an invisible digital watermarking technology based on modifying the edge pixels of text strokes to hide information in PDFs, which achieves high robustness to print–scan attacks. Moreover, it cannot be detected by human perception systems. This method focuses on the representation of text by embedding watermarks by changing the features of the text to ensure that changes in these features can be reflected in the scanned PDF after printing. We first segment each text line into two sub-blocks, then select the row of pixels with the most black pixels, and flip the edge pixels closest to this row. This method requires the participation of original PDF documents in detection. The experimental results show that all peak signal-to-noise ratio (PSNR) values of our proposed method exceed 32 dB, which indicates satisfactory invisibility. Meanwhile, this method can extract the hidden information with 100% accuracy under the JPEG compression attack, and has high robustness against noise attacks and print–scan attacks. In the case of no attacks, the watermark can be recovered without any loss. In terms of practical applications, our method can be applied in the practical leak tracing of official paper documents after distribution.

## 1. Introduction

PDF literally means portable document format. The advantages of PDF files are platform independence, small file size, good browsing quality, hyperlink placement, security setting, etc., so it has gradually become a mainstream medium, widely used in document transmission, exchange, and circulation, but its wide application also has many problems. For example, many published scientific articles are illegally and freely shared online in PDF format, posing a serious threat to the business model of academic publishers. In the Panama file, 2.2 million leaked files were in PDF format (email ranked first with 4.8 million leaked copies). In addition, PDF files can also be forged or fabricated, thereby questioning the integrity of PDF files. Thus, how to protect the security of PDF documents has become a prominent issue.

Invisible digital watermarking technology [1,2] is an important technology for achieving PDF protection [3]. This technology embeds hidden information in PDF files that cannot be detected by human perception systems. Only dedicated watermark detectors can detect or extract the embedded hidden information, which can then be used for various purposes, such as declaring ownership of PDF files (watermarks), tracking the illegal distribution of confidential documents (fingerprints), and checking if the document comes from another person (authentication), etc. [4]. PDF is a document format, so the PDF digital watermark is a document watermark. In recent years, researchers have proposed various PDF document watermarking algorithms, but they are still in a relatively early stage.

According to the application scope and algorithm characteristics, current PDF watermarking algorithms can be divided into the following categories: PDF digital watermarks for copyright protection and leak tracing, PDF digital watermarks for tamper detection and electronic signatures [5,6,7,8], PDF digital watermarks for secure communication [9,10,11,12,13,14,15,16,17], and multi-functional PDF digital watermarks [18,19,20]. The watermarking schemes belonging to the second category need to detect whether the PDF document has been tampered with. The main purpose of the third category is confidential communication. Users can hide information in PDF files through invisible watermarking technology during the production or compilation process, ensuring that hidden information is difficult to detect and not analyzed when viewing PDF files. As for the fourth category, the multi-functional PDF watermark technology is designed to implement watermarks with multiple characteristics simultaneously, allowing for a set of watermark schemes to achieve multiple application goals.

In this paper, we aim to propose a print–scan resistant PDF watermarking algorithm, which belongs to the first category. Due to copyright protection and leakage tracing requirements, information describing copyright or leakage traces can be embedded in PDF documents, which requires a large watermark capacity. At the same time, the requirements for information extraction ability, concealment, and robustness are high, and the content information of PDF documents cannot be modified. Grammar-based and semantic-based text watermarking techniques are not suitable; therefore, text watermarking algorithms based on document structure are generally chosen for copyright protection and leak tracing of PDF documents.

However, to achieve copyright protection and leak tracing of PDF documents after printing and distribution by utilizing digital watermarking technology, it is necessary to design specialized watermarking algorithms. The previous algorithms that utilize PDF file structural elements will no longer be applicable, as these structural attributes cannot be obtained through scanning after printing, making it challenging to extract watermarks. Therefore, efforts must be made in the form of text representation, studying how to embed watermarks by changing the features of the text, and ensuring that the changes in these features after printing can be reflected through the scanned PDF.

Based on the above considerations, we focus on the form of text representation and propose a scheme to modify the edge pixels of text strokes. The contributions of our paper are as follows:

1 We propose a novel digital watermarking technology in PDF documents based on modifying the edge pixels of text strokes. The proposed method solves the problem that conventional PDF structure-based PDF watermarking cannot resist print–scan attacks, which enables our method to be applied to the leak tracking of scanned versions of paper and PDF documents.
2 We propose a segmentation algorithm that can effectively avoid watermark extraction errors caused by asynchronous character segmentation during embedding and extraction. Meanwhile, the proposed segmentation method can change the strokes of multiple characters at once, so it has high robustness.
3 The proposed embedding algorithm has good invisibility, making it difficult to see traces of character modifications. Meanwhile, we guarantee that the color features of PDF files will not be affected after embedding the copyright information. Moreover, it is applicable to both Chinese and English.

## 2. Related Works

### 2.1. PDF Watermarking Technology for Copyright Protection and Leak Tracing

Many researchers introduced image digital watermarking technology in PDF documents, embedding binary watermark images in PDF documents to achieve copyright protection. This kind of algorithm has the characteristics of large information capacity and strong robustness. For example, they designed PDF watermarking algorithms based on Image Plus Acrobat, which embeds the matrix obtained from the watermark image into a PDF file and then uses the Acrobat PDF TouchUp tool to adjust character spacing and color to embed watermark information.

Furthermore, some researchers designed PDF digital watermarking algorithms based on document format. They often use space encoding and Arnold scrambling technology [21] to conduct Arnold scrambling transformation on the original watermark image, embed the transformed binary image watermark into the line composed of spaces inserted in the post script (PS) file corresponding to the PDF document, and then convert the PS file into a PDF file. The experiment shows that this kind of algorithm has good invisibility and robustness.

Many researchers choose the structure of PDF documents as a feature to embed the watermark. In [22], the authors use the discarded page object of a PDF to embed the watermark. Although this scheme has the advantages of good concealment, robustness, and simple implementation, this method is not widely applicable because many PDF documents that are modeled once do not have discard objects. Zhang et al. [23] also propose a PDF document watermarking algorithm based on discarded page objects. This method can tolerate deleting some words, adding some words, and even allowing certain pages to be deleted. The embedded watermark can be increased or decreased at any time, so the hiding capacity is large. The simulation results show that the algorithm is simple to implement and has strong robustness. Some researchers analyzed the structure of PDF documents in depth and designed PDF watermarking methods based on modified font saturation and rendering mode. This kind of method achieves high invisibility and robustness, has a large watermark information capacity, and achieves identification against third-party software tampering attacks based on detecting gray-scale values that cannot be modified by third parties.

In addition, many researchers specifically considered the issue of large-capacity PDF watermarking schemes increasing document size and designed watermarking schemes that modify the document structure. This kind of algorithm utilizes the invisibility of line-end identifiers in PDF documents and embeds the watermark by equivalently replacing each line-end identifier in cross-referencing tables. This kind of algorithm has a large information capacity, good concealment, and can resist statistical attacks and other attack methods. Shaikhli et al. [24] proposed a PDF watermarking algorithm that inserts digital signatures as invisible watermarks. This algorithm utilizes the feature that the space characters of the segmented fields in the cross-reference table can be overwritten without damaging the file to embed watermarks in PDF files. The algorithm does not change the size of the document.

### 2.2. Print–Scan-Resilient Watermarking Schemes

Although some PDF watermarking technologies were proposed for copyright protection and leak tracing, few of these methods are resistant to print–scan attacks, which is a huge threat to the copyright protection of printed matters. Most prior works on print–scan-resilient watermarking schemes are for color and gray-scale images, in which the pixels take on a wide range of values [25,26,27,28,29,30,31]. However, such methods are not suitable for PDF files since not all PDF files have color images or gray-scale images. Some watermarking schemes against print–scan attacks were proposed for text images [32,33,34,35,36]. Wu and Liu [35] developed a watermarking method by manipulating flippable pixels based on specific block-based relationships. Although this method achieved high watermark capacity, it has high requirements for printing and scanning quality to extract watermarks, so its robustness against the print–scan process is not satisfactory. Tan et al. [33] proposed a feature-based text image watermarking scheme against the print–scan process. A mathematical multiplicative transformation model was employed to approximate geometrically invariant features that survive the print–scan process and thus serve as reference points for watermark embedding and extraction. However, we experimentally demonstrated that this method is poorly robust in practice.

The PDF file contains a large amount of text content, and it may also contain images, hyperlinks, and other elements. Moreover, a PDF file may contain text content in different font sizes and typefaces, but most existing schemes do not support embedding information in multiple fonts at the same time, such as the scheme proposed in [33]. Thus, the above-mentioned watermarking schemes cannot be directly utilized for protecting PDF files.

## 3. Proposed PDF Watermarking Algorithm Resistant to Print–Scan Attack

### 3.1. Overall Idea

In order to utilize digital watermarking technology to achieve leak tracing of PDF documents after printing and distribution, it is necessary to design specialized watermarking algorithms. The previous algorithms that utilize PDF file structural elements will no longer be applicable, as these structural attributes cannot be obtained through scanning after printing, making it difficult to extract watermarks. Therefore, efforts must be made in the form of text representation, studying how to embed watermarks by changing the features of the text, and ensuring that the changes in these features after printing can be reflected through the scanned PDF.

**Figure 1:**
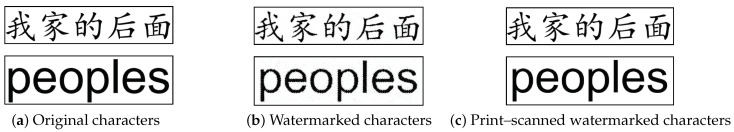
Comparison of original characters, watermarked characters and print–scanned watermarked characters. (**a**) (**top**): Chinese characters meaning “the back of my house”, (**bottom**): English characters; (**b**) watermarked characters corresponding to (**a**); (**c**) printed-scanned watermarked characters corresponding to (**a**).

Based on the above considerations, this paper proposes a scheme to modify the edge pixels of text strokes. Figure 1 provides an example of modifying the stroke edge pixels of the text in Figure 1a to obtain Figure 1b, which can be observed when zoomed in. After printing and scanning, Figure 1c can still be observed even when zoomed in. Therefore, modifying the edge pixels of text strokes is a feasible solution.

The issues that need to be considered in designing our algorithm framework include the following aspects:

(1) How to accurately cut the text in document images?
(2) Which strokes are selected for modification?
(3) How to compare and extract watermark information during extraction?

In response to problem (1), we found that after the binarization of PDF document images, a single text can be roughly cut using row and column projection methods. The gaps between individual text will not change after embedding the watermark and before embedding the watermark. Then, we utilized this feature to divide each row into two sub-blocks, with the segmentation point being the location with the largest text spacing. Compared with segmenting a single character, splitting a text line into two sub-blocks is more robust because it avoids the accumulation of extraction errors due to single character segmentation errors. Our approach to problem 2 is to select the longest stroke to modify the surrounding edge pixels, which can improve the invisibility and robustness of the watermark. Considering that in the application system for distributing official documents, the original PDF electronic document before printing can be obtained, our extraction scheme is designed as non-blind extraction, which not only reduces the difficulty of embedding the algorithm design but also improves the success rate of detection. However, the noise generated in the print–scan process will make the position of the watermarked sub-block relative to the original sub-block higher or lower. To deal with this problem, we propose an adaptive alignment model to automatically adjust the position of the watermarked image. Then, we compare the specific pixels selected at the embedding stage and then extract information according to the comparison, which will be described in detail in the following.

### 3.2. Embedding Algorithm

**Figure 2:**
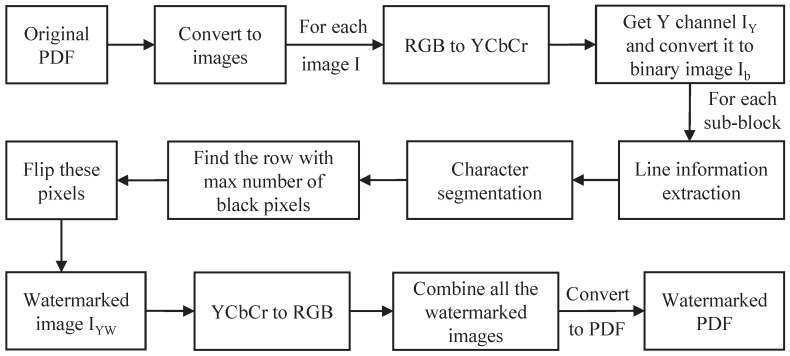
Proposed watermark embedding scheme.

Based on above overall idea, the embedding process of the proposed PDF watermarking algorithm is shown in Figure 2 and the corresponding embedding algorithm is shown in Algorithm 1. The detailed process can be illustrated as follows:

(1) Step 1: Convert the original PDF file into a series of images, and perform subsequent operations on each image.

(2) Step 2: Map the original image I from the RGB color space to the YCbCr color space, decompose the Y component, mark it as $I_{Y}$, and convert it into a binary text image $I_{b}$;

(3) Step 3: Perform character segmentation on the binary text image $I_{b}$, and the segmentation process is as follows:

(1) Step 3.1: Firstly, perform line information extraction. The basic idea of line information extraction is to divide character blocks based on the characteristics of the connected domain of characters. For $I_{b}$, the white background is 1, and the black text is 0. Then, the sum of pixels is calculated by line, and the area with a cumulative sum of 0 is classified as the text area. Considering text connectivity, the number of lines *P* and the upper and lower boundaries of each line can be obtained based on the divided area.

(2) Step 3.2: Then, for each text line, sum the column pixels separately, and further confirm the left and right boundaries of each text line based on character connectivity. Next, calculate the spacing between each two characters and select the maximum spacing to divide the current line into two sub-blocks, that is, each line is divided into two sub blocks, so there are a total of K=2P sub-blocks.

(3) Step 3.3: Repeat operations 3.1 to 3.2 to obtain the set of sub-blocks $\left\{B_{i}|i=1,2,\ldots ,K,K=2P\right\}$ to embed the watermark. $B_{i}$ contains $r_{i}$ rows and $c_{i}$ columns of pixels (note: the $r_{i}$ and $c_{i}$ of each $B_{i}$ may not be the same).

(4) Step 4: Assuming that the watermark bit sequence to be embedded is $w_{1},w_{2},\ldots ,w_{N}$, if N>K, an error exit will be prompted. Otherwise, modify $B_{i}$ to ${\hat{B}}_{i}$ based on $w_{i}$ being 0 or 1, and the modification process is as follows:

(1) Step 4.1: Calculate the number of black pixels in each row of $B_{i}$ and find the row with the highest number of black pixels, denoted as $r_{long}$;

(2) Step 4.2: Extract the columns with black pixels from $r_{long}$ and mark them as $L=l_{1},l_{2},\ldots ,l_{n}$, $n<c_{i}$;

(3) Step 4.3: For each column in *L*, extract all pixels of that column, find the white pixel closest to $r_{long}$, and mark it as $p=I_{b}\left(x,y\right)$. When the watermark bit to be embedded is ‘1’, first determine the position of *p*. If *p* is above $r_{long}$, flip the pixel in $I_{Y}$ with position (x,y+1) to a white pixel; When *p* is located below $r_{long}$, flip the pixel in $I_{Y}$ with position (x,y−1) to a white pixel. When the watermark bit to be embedded is ‘0’, flip the pixel at position (x,y) in $I_{Y}$ to black pixels. Thus, we obtain the watermarked image $I_{\mathrm{YW}}$.

(4) Step 4.4: Replace the *Y* channel in the YCbCr color space with $I_{\mathrm{YW}}$, map it back to RGB space, and save the watermarked image $I_{W}$.

(5) Step 5: After embedding all images, combine all watermarked images in the original order to generate a PDF file with watermarks.
**Algorithm 1** Embedding algorithm**Input:** Original PDF file, watermark bit sequence $w_{1},w_{2},\ldots ,w_{N}$**Output:** Watermarked PDF file1:Convert the original PDF file into a series of images $I_{1},\ldots ,I_{n}$2:**for** each image I **do**3:   Map I from the RGB color space to the YCbCr color space, decompose the Y component, mark it as $I_{Y}$, and convert it into a binary text image $I_{b}$.4:   Perform character segmentation and text line information extraction.5:   **for** each text line **do**6:     Confirm the left and right boundaries of each text line.7:     Calculate the spacing between each two characters and select the maximum spacing to divide the current line into two sub-blocks.8:   **end for**9:   Obtain the set of sub-blocks $\left\{B_{i}|i=1,2,\ldots ,K,K=2P\right\}$, *P* is the number of text lines.10:  **for** each sub-block $B_{i}$ **do**11:     Calculate the number of black pixels in each row of $B_{i}$ and find the row with the highest number of black pixels, denoted as $r_{long}$.12:     Extract the columns with black pixels from $r_{long}$ and mark them as $L=l_{1},l_{2},\ldots ,l_{n}$, $n<c_{i}$.13:     **for** each column in *L* **do**14:        Extract all pixels of that column, find the white pixel closest to $r_{long}$, and mark it as $p=I_{b}\left(x,y\right)$.15:        **if** $w_{i}$ is “1” **then**16:          When *p* is above $r_{long}$, flip the pixel in $I_{Y}$ with position (x,y+1) to a white pixel; When *p* is located below $r_{long}$, flip the pixel in $I_{Y}$ with position (x,y−1) to a white pixel.17:        **end if**18:        **if** $w_{i}$ is “0” **then**19:          Flip the pixel at position (x,y) in $I_{Y}$ to black pixels.20:        **end if**21:     **end for**22:   **end for**23:   Get the watermarked image $I_{\mathrm{YW}}$.24:   Replace the *Y* channel in the YCbCr color space with $I_{\mathrm{YW}}$, map it back to RGB space.25:   Save the watermarked image $I_{W}$.26:**end for**27:Combine all watermarked images in the original order and convert to a PDF file with watermarks.

### 3.3. Extraction Algorithm

**Figure 3:**
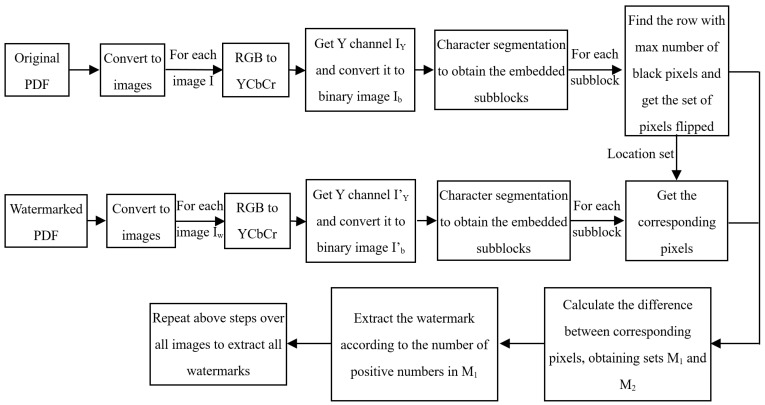
Proposed watermark extraction scheme.

The extraction algorithm requires the participation of the original PDF file and is a non-blind detection algorithm. The extraction process of the proposed PDF watermarking algorithm is shown in Figure 3 and the corresponding extraction algorithm is shown in Algorithm 2. The detailed process is as follows.

(1) Step 1: Convert the original PDF file and the test PDF file into a series of images, and perform subsequent operations on each original image I and the test image $I_{W}$;

(2) Step 2: Correct the geometric distortions and remove the isolated noise for $I_{W}$. Map the image I and $I_{W}$ to be detected from RGB space to YCbCr space, decompose the Y components, mark it as $I_{Y}$ and $I_{Y}^{\prime }$, and convert them into binary text images $I_{b}$ and $I_{b}^{\prime }$.

(3) Step 3: Perform character segmentation on the binary text image $I_{b}^{\prime }$ using the same method as when embedding the watermark. After segmentation, obtain the set of sub blocks $\left\{B_{i}^{\prime }|i=1,2,\ldots ,K,K=2P\right\}$ for the watermark to be extracted, where *P* is the number of rows.

(4) Step 4: Similarly, perform the above operation on the original binary image $I_{b}$ to obtain the set $\left\{B_{i}|i=1,2,\ldots ,K,K=2P\right\}$.

(5) Step 5: For each $B_{i}$, adaptively adjust its corresponding $B_{i}^{\prime }$ to ensure that the pixels of $B_{i}$ and $B_{i}^{\prime }$ correspond one by one to prevent watermark extraction failure due to pixel offset. The adaptive adjustment process is as follows:

First, calculate the similarity between $B_{i}^{\prime }$ and $B_{i}$ using the following formula.

(1) $$\mu_{i}^{\left(1\right)}=\frac{\sum_{p=0}^{r_{i}}\sum_{q=0}^{c_{i}}{\left(B_{i}^{\prime }\left(p,q\right)-B_{i}\left(p,q\right)\right)}^{2}}{r_{i}\times c_{i}}$$

Afterwards, remove the leftmost column pixels of $B_{i}^{\prime }$ and add one row of pixels to the rightmost column, with pixel values of 255. Use Formula (1) to calculate the similarity $\mu_{i}^{\left(2\right)}$ between $B_{i}^{\prime }$ and $B_{i}$ at this time.

Similarly, remove the rightmost column pixels of $B_{i}^{\prime }$ and add one row of pixels to the leftmost column, with pixel values of 255. Use Formula (1) to calculate the similarity $\mu_{i}^{\left(3\right)}$ between $B_{i}^{\prime }$ and Bi at this time.

Similarly, remove the first row of pixels from $B_{i}^{\prime }$ and add one row of pixels below the ri row of $B_{i}^{\prime }$, with pixel values of 255. Use Formula (1) to calculate the similarity $\mu_{i}^{\left(4\right)}$ between $B_{i}^{\prime }$ and Bi at this point.

Similarly, remove the pixels in the $r_{i}$ th row of $B_{i}^{\prime }$ and add a row of pixels above the first row of $B_{i}^{\prime }$, with pixel values of 255. Use Formula (1) to calculate the similarity $\mu_{i}^{\left(5\right)}$ between $B_{i}^{\prime }$ and $B_{i}$ at this time.

Compare the values of $\mu_{i}^{\left(1\right)}-\mu_{i}^{\left(5\right)}$ and select the $B_{i}^{\prime }$ corresponding to the minimum $\mu_{i}=\min\left\{\mu_{i}^{\left(1\right)},\mu_{i}^{\left(2\right)},\mu_{i}^{\left(3\right)},\mu_{i}^{\left(4\right)},\mu_{i}^{\left(5\right)}\right\}$ for the following operation.

(6) Step 6: Calculate the number of black pixels in each row of $B_{i}$, find the row with the highest number of black pixels, and mark it as $r_{long}$.

(7) Step 7: Extract the columns with black pixels from $r_{long}$ and mark them as $L=\left\{l_{1},l_{2},\ldots ,l_{n}\right\},n<c_{i}$.

(8) Step 8: For each column in *L*, extract all the pixels in that column, find the white pixel closest to $r_{long}$, and mark it as $I_{b}\left(x,y\right)$. Extract pixel $I_{b}^{\prime }\left(x,y\right)$ from the watermarked image $I_{b}^{\prime }$, and calculate the difference $m_{1}^{\left(i\right)}$ between $I_{b}^{\prime }\left(x,y\right)$ and $I_{b}\left(x,y\right)$.

(9) Step 9: Repeat steps 6–8 to obtain sets $M_{1}=\left\{m_{1}^{\left(i\right)}|i=1,2,\ldots ,N\right\}$. Calculate the number of positive and negative numbers in $M_{1}$. If the number of positive numbers and 0s in $M_{1}$ is greater than the number of negative numbers, denote as $M_{1}^{\left(+\right)}$, and otherwise denote as $M_{1}^{\left(-\right)}$.

(10) Step 10: If $M_{1}=M_{1}^{\left(+\right)}$, the extracted watermark bit is ‘1’, and if $M_{1}=M_{1}^{\left(-\right)}$, the extracted watermark bit is ‘0’.

(11) Step 11: Repeat steps 5–10 to extract all watermark bits and obtain the watermark string (length *N*) extracted from the current image.

(12) Step 12: Overlay and average all extracted watermark strings to obtain the final extracted watermarks $w_{1}^{\prime },w_{2}^{\prime },\ldots ,w_{N}^{\prime }$.

## 4. Experiment and Analysis

This section will evaluate the anti-print–scan PDF watermarking algorithm proposed in this paper in various aspects, including imperceptibility and the ability to resist various attacks (including JPEG compression, adding Gaussian noise, adding salt–pepper noise, scaling, various filtering attacks, and experiencing print–scan operations). We used a total of five PDF test documents, each containing three pages. In order to test the applicability of the proposed algorithm, the contents of five documents are listed in Table 1.
**Algorithm 2** Extraction algorithm**Input:** Original watermarked PDF file and watermarked PDF file**Output:** Extracted watermark sequence1:Convert the original PDF file and the watermarked PDF file into a series of images, and perform subsequent operations on each original image I and the watermarked image $I_{W}$. Map the image I and $I_{W}$ to be detected from RGB space to YCbCr space, decompose the Y components, mark it as $I_{Y}$ and $I_{Y}^{\prime }$, and convert them into binary text images $I_{b}$ and $I_{b}^{\prime }$.2:**for** each original image $I_{b}$ and corresponding watermarked image $I_{b}^{\prime }$ **do**3:   Obtain the set of sub-blocks $\left\{B_{i}|i=1,2,\ldots ,K,K=2P\right\}$, and $\left\{B_{i}^{\prime }|i=1,2,\ldots ,K,K=2P\right\}$, where *P* is the number of rows.4:   **for** each $B_{i}$ **do**5:     Adjust its corresponding $B_{i}^{\prime }$ to ensure that the pixels of $B_{i}$ and $B_{i}^{\prime }$ correspond one by one.6:     Calculate the number of black pixels in each row of $B_{i}$, find the row with the highest number of black pixels, and mark it as $r_{long}$.7:     **for** each column in *L* **do**8:        Find the white pixel closest to $r_{long}$, and mark it as $I_{b}\left(x,y\right)$.9:        Extract pixel $I_{b}^{\prime }\left(x,y\right)$ from the watermarked image $I_{b}^{\prime }$.10:      Calculate the difference $m_{1}^{\left(i\right)}$.11:    **end for**12:    Obtain sets $M_{1}=\left\{m_{1}^{\left(i\right)}|i=1,2,\ldots ,N\right\}$.13:    Extract watermark bit according to the number of positive and negative numbers in $M_{1}$.14:  **end for**15:  Extract all watermark bits and obtain the watermark string (length *N*) extracted from the current image.16:**end for**17:Overlay and average all extracted watermark strings to obtain the final extracted watermarks $w_{1}^{\prime },w_{2}^{\prime },\ldots ,w_{N}^{\prime }$.

### 4.1. Invisibility

**Figure 4:**
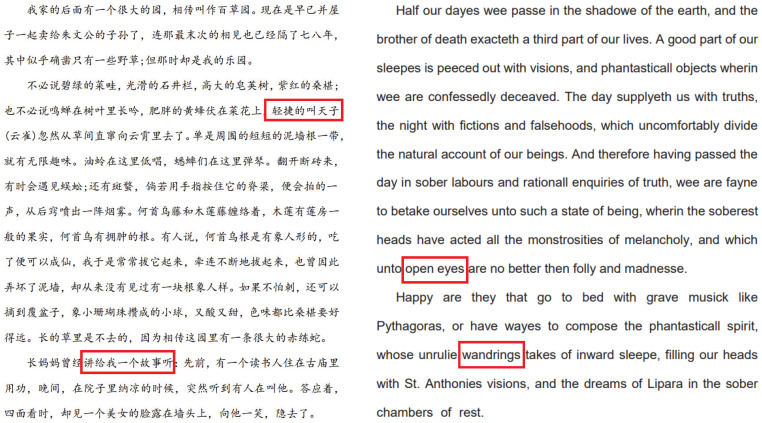
Original document images. (**Left**) Chinese article. (**Right**) English article.

**Figure 5:**
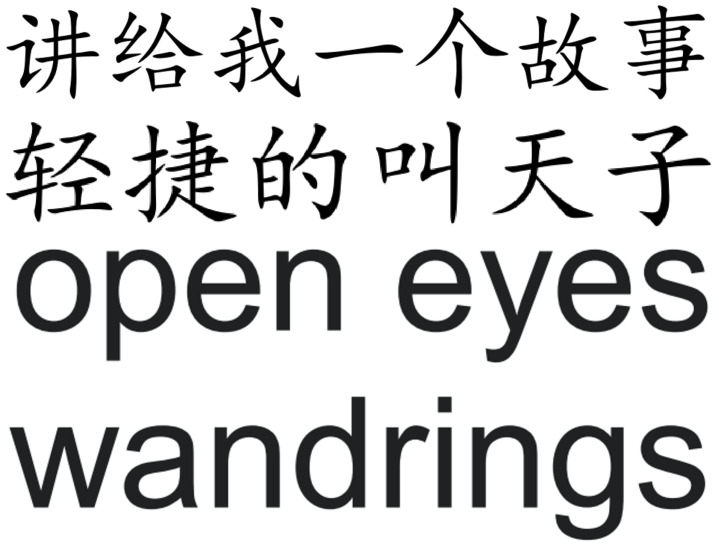
Enlarged characters from original document images. First and second line: Chinese characters selected from the Chinese article in the left of Figure 4, third and forth line: English characters selected from the English article in the right of Figure 4.

**Figure 6:**
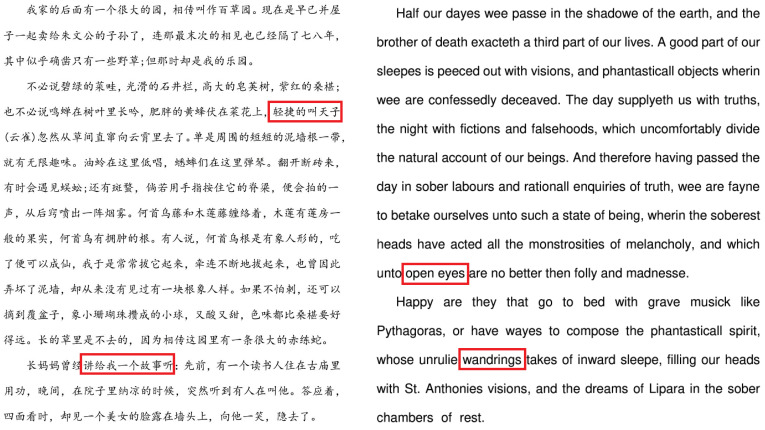
Watermarked document images. (**Left**) watermarked Chinese article corresponding to the Chinese article in the left of Figure 4, (**Right**) watermarked English article corresponding to the English article in the right of Figure 4.

**Figure 7:**
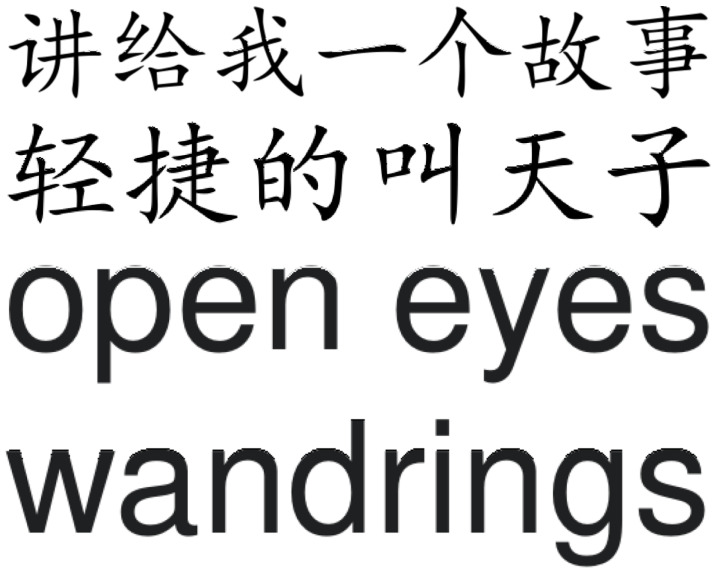
Enlarged characters from watermarked document images. First and second line: watermarked Chinese characters selected from the Chinese article in the left of Figure 6, third and forth line: English characters selected from the English article in the right of Figure 6.

This section uses PSNR to measure the invisibility of the algorithm. Figure 4 shows two pages selected from our experimental PDF files. Two pages selected from Figure 5 display the enlarged part of the characters with red blocks in Figure 4. The images after embedding the watermark are shown in Figure 6, and Figure 7 displays the enlarged part of the characters with red blocks in Figure 6. A careful comparison between Figure 5 and Figure 7 shows that there is no noticeable degradation in the visual effect of the watermarked image, and it is almost impossible to see that the characters have undergone changes. In fact, the modification of characters only reflects changes in edge pixels, without changing the overall effect of the characters. From Table 2, it can be seen that the average PSNR of the measured documents and the PSNR values of each page of the documents both exceed 32 dB, indicating that the imperceptibility of this scheme is very good. Table 3 gives the comparison of the proposed framework with [33,35] in terms of the average PSNR values.

### 4.2. Robustness

While there have been numerous research efforts in detecting and applying specific preprocessing techniques to enhance robustness against different types of attacks [37], our method achieves robustness against various attack types without requiring attack detection or corresponding preprocessing. When testing robustness, a 32-bit binary sequence was repeatedly embedded three times (once per page) in each document’s three pages as watermark. We first extract the watermark of each page in the PDF file, and then obtain the final PDF watermark according to the mechanism of minority obeying the majority. For example, if a PDF file has 3 pages, and the first watermark bits we extract from each page are 0, 0, 1 respectively, then the first bit of the PDF watermark is 0. The number of error bits is utilized to evaluate robustness, with a smaller number indicating higher robustness, while a larger number indicates lower robustness.

#### 4.2.1. JPEG Compression Attack Testing

This section tests the ability of the proposed algorithm to resist JPEG compression. JPEG compression is a common image attack method. Some pixels of the image are lost during JPEG compression. In our experiment, it will especially affect the edge pixels of the text, which is not conducive to watermark extraction. We embed watermarks on a total of 15 pages of content in five documents and then compress them with JPEG before performing watermark extraction. The JPEG compression quality factor (QF) varies from 10 to 90 with an interval of 10. As shown in Table 4, each QF has two sub-columns. The first sub-column represents the number of 32-bit watermark extraction errors in the three pages of each PDF file, and the second sub-column represents the number of error bits after the watermark redundancy judgment of the three pages according to the principle minority obeying majority. From Table 4, it can be seen that although there may be errors in single-page extraction, three-page redundant extraction ensures no errors.

#### 4.2.2. Noise Attack

This section tests the robustness of the proposed algorithm under Gaussian noise and salt–pepper noise attacks. The text images embedded with watermarks were subjected to salt–pepper noise and Gaussian noise with intensities of 0.001 and 0.01, respectively. As shown in Table 5, each kind of attack has two sub-columns. The first sub-column represents the number of 32-bit watermark extraction errors in the three pages of each PDF file, and the second sub-column represents the number of error bits after redundancy judgment is performed on the watermarks extracted from the three pages according to the principle of minority obeying the majority. The results of Table 5 indicate that the proposed algorithm has high robustness against salt-pepper noise, and can accurately extract watermarks under salt-pepper noise with both 0.01 and 0.001 intensities. However, when the proposed algorithm is applied to English documents, its robustness to Gaussian noise is lower than that of Chinese documents or mixed Chinese and English documents. We can observe from Table 5 that when the document is in full English (PDF4), the number of error bits of a single page is higher than others, and there is a 1-bit extraction error of the whole PDF4 under Gaussian noise with intensities of 0.01 and 0.001.

#### 4.2.3. Scaling Attack

In this section, we verify the robustness against the scaling attack of our proposed method. The width and height of watermarked images are scaled proportionally with the scaling factor from 0.75 to 2.0. As shown in Table 6, each scaling factor has two sub-columns. The first sub-column represents the number of 32-bit watermark extraction errors in the three pages of each PDF file, and the second sub-column represents the number of error bits after redundancy judgment is performed on the watermarks extracted from the three pages according to the principle of minority obeying the majority. In Table 6, the proposed scheme can recover the hidden information almost without error under all scaling factors. Especially when the scaling factor is 2, the difference between the watermark sub-block and the original sub-block is amplified, so the number of error bits is much lower than others.

#### 4.2.4. Filtering Attack

Different filters of size 3×3 are applied to the watermarked images, and the BEQ values are recorded in Table 7. We can observe that each kind of filter has two sub-columns. The first sub-column represents the number of 32-bit watermark extraction errors in the three pages of each PDF file, and the second sub-column indicates the number of error bits after redundancy judgment is performed on the watermarks extracted from the three pages according to the principle of minority obeying the majority. The proposed watermarking scheme can recover the message almost without error under all 3×3 filtering attacks. Specifically, the PDF3 is more vulnerable to three filtering attacks than other PDF files. The basic reason is that the PDF3 file contains some words of size 10.5. In this case, the number of flipping pixels is less than others, which is more susceptible to noise interference during printing and scanning.

#### 4.2.5. Print–Scan Attack

This section tests the robustness of the watermarked PDF files against print–scan attacks. The specific process is to use a regular desktop printer (HP LasarJet Pro M17w with resolution 600 DPI) to print the watermarked PDF files. Subsequently, the printed watermarked PDF is scanned into a PDF file in color image format using a regular scanner (EPSON PER-FECTION V30 SE with resolution 600 DPI). Then we use the watermark extraction steps in the previous section to extract the watermark. In Table 8, we document the extraction results of our proposed method with and without the print–scan attack. Both “no attack” and “print–scan attack” have two sub-columns. The first sub-column represents the number of 32-bit watermark extraction errors in the three pages of each PDF file, and the second sub-column represents the number of error bits after redundancy judgment is performed on the watermarks extracted from the three pages according to the principle of minority obeying the majority. From Table 8, it can be seen that the method proposed in this paper has good robustness against print–scan attacks, and the number of error bits is within an acceptable range. As for PDF2, we can observe that although PDF2 and PDF4 have similar error rates per page, PDF2 can achieve error-free extraction after the minority obeys the majority mechanism. It should be noted that the robustness of the algorithm in this paper for all English documents is lower than that of Chinese documents. We make the following explanation here. Due to the characteristics of Chinese characters, when we perform left and right segmentation on the current line, we extract the line with the highest number of black pixels and modify it around it. Chinese characters have the majority of strokes, and those with “one horizontal” characters have higher robustness than other Chinese characters. However, this feature is missing in English, where only the letters “e”, “f”, and “t” have “one horizontal”. Therefore, when we modify English, we can only modify some vertices or strokes with radians, but these strokes are prone to changes after print–scan operations (prone to stacking noise), so the robustness of English is lower than that of Chinese.

### 4.3. Comparison with Existing Print–Scan Schemes

We also compare our proposed method with state-of-the-art works in terms of performance against the print–scan process, print–copy(1)–scan, print–copy(1)–scan, JPEG compression, Gaussian filter, and salt–pepper noise. In Section 2, we mention that most PDF-based watermarking technologies for copyright protection and leak tracing are not resistant to print–scan attacks. Thus, to test the robustness against print–scan, we choose two print–scan resilient watermarking schemes which are image-based for comparative experiments. A 32-bit watermark sequence is repeatedly embedded three times (once per page) in each document’s three pages using three watermarking schemes, and the number of error bits after three-page redundancy is recorded in Table 9. We can observe that our method is much more robust against the print–scan attack than the other two methods. The following analyzes the possible reasons for the extraction failure: the experimental equipment (printers and scanners) used in [35], [33] and this paper is different. For example, the highest resolution of the printer used in this paper is only 600 DPI, but the highest resolution of the printer in [33] can achieve 1200 DPI. A lower resolution indicates that more noise will be introduced into the paper document, which will result in the inconsistent segmentation of individual characters when embedding and extracting. In addition, refs. [33,35] rely on the ratio of black pixels to all pixels in each embedding unit to embed information, but too much noise will break this relationship. However, our proposed scheme still performs well on printers with a resolution of 600 DPI, let alone high-resolution printers. For the print–copy–scan operation, as the number of copies increases, our method exhibits an increasing number of errors. This is due to the accumulation of noise introduced with each copy, leading to greater distortion in the textual image.

For the print–copy–scan operation, as the number of copies increases, our method exhibits an increasing number of errors. This is due to the accumulation of noise introduced with each copy, leading to greater distortion in the textual image. However, we can observe that for the two methods in [33,35], the increase in the number of copies does not affect their error bits. This is because they lack sufficient robustness to withstand print–scan attacks, let alone print–copy–scan operations. Therefore, for both bit values 0 and 1, these two methods have a probability of 0.5 to extract the correct value. As a result, we can see that the error bits generally fluctuate around 16 bits.

All methods achieve strong robustness against JPEG compression when quality factor is 10. As for salt–pepper noise, when the factor is 0.01 and with the scaling attack, the performance of the proposed scheme is similar to that of [33] and better than that of [35]. The robustness against the Gaussian filter of [35] and our proposed scheme is comparable, but the robustness of [33] is somewhat lower.

### 4.4. Time Complexity

**Figure 8:**
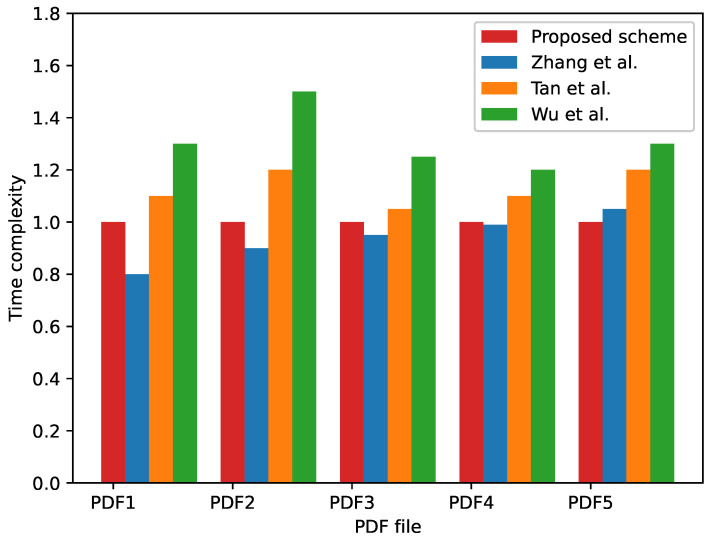
Comparison of time complexity with existing methods. Blue: method in [23], orange: method in [33], green: method in [35].

In this section, we compare the proposed method with three existing watermarking methods in terms of the runtime of embedding methods. One of the methods is a PDF watermarking algorithm based on the PDF structure [23], while the other two methods are print–scan-resilient watermarking algorithms based on text images. The comparison is performed using a multiplier relationship, where the different colors of bars in Figure 8 represent the ratio of the runtime of comparative method to the runtime of the proposed method.

When the ratio is less than 1, it indicates that the comparative method is faster than our method. The smaller the ratio, the faster the compared method relative to our method. When the ratio is greater than 1, it indicates that the comparative method is slower than our method. The larger the ratio, the slower the compared method relative to our method. From Figure 8, we can observe that the runtime of our method and the method in [23] are comparable, and both of them are faster than the other two methods [33,35].

## 5. Conclusions

This paper proposes a PDF watermarking algorithm based on modifying character stroke edge pixels that can resist one-time print–scan attacks. This method requires the participation of the original PDF documents in detection and is robust against noise attacks, compression attacks, and print–scan attacks. It can be applied to the leak tracing of official paper documents after distribution. From the algorithm steps and simulation experimental results, it can be seen that the advantages of this scheme are as follows: (1) The segmentation algorithm proposed in this paper can effectively avoid watermark extraction errors caused by asynchronous character segmentation during embedding and extraction. (2) The segmentation method proposed in this paper can change the strokes of multiple characters at once, so it has high robustness. (3) The embedding algorithm proposed in this paper has good invisibility, making it difficult to see traces of character modifications. From the algorithm steps and simulation experimental results, it can be seen that the drawbacks of this scheme are as follows: (1) Currently, only non-blind extraction is supported. To achieve blind extraction, the extractor needs to generate a file with the same format as the watermark file for extraction. (2) Embedded capacity has certain limitations. Currently, only 2-bit information can be embedded in a single line of documents. In the future, how to realize the blind extraction of the current work is one of our considerations.

## Figures and Tables

**Table 1:** The contents of five experimental documents.

PDF Document	Language	Font	Size	Have Image?
PDF1	Chinese	Song	14 pt	No
PDF2	Chinese & English	Song & Arial	14 pt	No
PDF3	Chinese	Song	14 pt & 10.5 pt	Yes
PDF4	English	Arial	14 pt	No
PDF5	Chinese	KaiTi	14 pt	No

**Table 2:** PSNR of PDF images after embedding watermarks.

PDF Document	Each PSNR (dB)	Average PSNR (dB)
PDF 1	37.56	37.22
	36.59	
	37.52	
PDF 2	34.81	34.50
	34.43	
	34.28	
PDF 3	34.62	34.74
	34.64	
	34.97	
PDF 4	33.25	33.04
	32.83	
	33.06	
PDF 5	34.04	33.90
	33.85	
	33.83	

**Table 3:** Comparison with existing works in terms of PSNR values.

PDF Document	Wu et al. [35]	Tan et al. [33]	Ours
PDF1	31.16	34.52	37.22
PDF2	29.58	33.29	34.50
PDF3	28.74	32.78	34.74
PDF4	29.45	31.44	33.04
PDF5	30.11	32.69	33.90

**Table 4:** Test results of JPEG compression attack.

QF	90		80		70		60		50		40		30		20		10	
PDF 1	0	0	1	0	1	0	1	0	1	0	0	0	0	0	0	0	1	0
	1		3		2		2		4		5		5		5		4	
	0		0		0		0		0		0		0		1		0	
PDF 2	0	0	0	0	0	0	0	0	0	0	0	0	0	0	0	0	0	0
	1		1		2		2		2		2		2		3		2	
	0		0		0		0		0		0		0		0		2	
PDF 3	0	0	1	0	0	0	0	0	1	0	1	0	1	0	0	0	2	0
	0		0		0		0		1		1		0		0		2	
	0		0		0		0		0		0		0		0		0	
PDF 4	4	0	4	0	4	0	4	0	4	0	4	0	5	0	5	0	7	0
	2		2		2		2		2		2		3		3		4	
	0		0		0		0		0		0		0		0		0	
PDF 5	2	0	1	0	1	0	0	0	0	0	0	0	0	0	0	0	0	0
	1		2		2		2		1		1		1		0		0	
	1		0		0		0		0		0		0		0		0	

**Table 5:** Test results of salt–pepper noise and Gaussian noise attacks.

Attack	Guassian 0.001		Guassian 0.01		Salt-Pepper 0.001		Salt-Pepper 0.01	
PDF 1	0	0	0	0	0	0	0	0
	2		2		1		1	
	0		0		0		0	
PDF 2	0	0	0	0	1	0	1	0
	1		2		0		1	
	1		2		0		0	
PDF 3	0	0	2	0	0	0	1	0
	0		0		0		1	
	0		0		0		0	
PDF 4	6	1	6	1	5	0	4	0
	3		3		2		3	
	2		2		0		0	
PDF 5	0	0	0	0	0	0	1	0
	1		0		0		0	
	0		0		0		0	

**Table 6:** Test results of scaling attacks.

Attack	Factor = 0.75		Factor = 1.5		Factor = 1.75		Factor = 2	
PDF 1	3	1	1	0	1	0	1	0
	3		0		0		0	
	4		0		0		0	
PDF 2	5	2	2	1	3	1	3	1
	4		3		2		2	
	3		2		1		2	
PDF 3	5	3	4	1	3	0	0	0
	5		2		2		0	
	4		2		1		0	
PDF 4	2	1	2	1	2	1	1	0
	3		2		2		1	
	2		4		2		0	
PDF 5	2	2	2	1	0	0	0	0
	4		5		3		0	
	3		1		0		0	

**Table 7:** Test results of different filter attacks.

Attack	Median Filter 3×3		Gaussian Filter 3×3		Average Filter 3×3	
PDF 1	1	0	2	0	2	0
	2		2		2	
	0		1		1	
PDF 2	3	0	2	0	2	0
	3		3		3	
	0		0		0	
PDF 3	2	1	2	1	2	1
	3		3		3	
	1		1		1	
PDF 4	2	0	2	0	2	0
	3		3		3	
	2		2		2	
PDF 5	1	0	1	0	1	0
	0		0		0	
	1		1		1	

**Table 8:** Test results of no attack and one-time print–scan attack.

PDF Document	No Attack		Print–Scan Attack	
PDF 1	0	0	3	3
	1		5	
	0		5	
PDF 2	0	0	8	0
	1		4	
	0		7	
PDF 3	0	0	3	1
	0		5	
	0		4	
PDF 4	1	0	8	4
	1		7	
	0		2	
PDF 5	0	0	5	3
	0		7	
	0		4	

**Table 9:** Comparison with existing works in terms of print–scan attack, JPEG compression, Gaussian filter, scaling and salt–pepper noise.

Attack	PDF	Wu et al. [35]	Tan et al. [33]	Ours
Print–scan	PDF1	20	15	3
	PDF2	15	13	0
	PDF3	17	14	1
	PDF4	13	16	4
	PDF5	16	10	3
Print-copy(1)-scan	PDF1	18	15	7
	PDF2	19	13	2
	PDF3	18	14	4
	PDF4	19	16	5
	PDF5	22	10	6
Print-copy(2)-scan	PDF1	21	15	10
	PDF2	19	13	15
	PDF3	15	14	9
	PDF4	15	16	12
	PDF5	13	10	10
JPEG compression 10	PDF1	0	0	0
	PDF2	0	0	0
	PDF3	0	0	0
	PDF4	0	0	0
	PDF5	0	0	0
Salt-Pepper noise 0.01	PDF1	9	0	0
	PDF2	5	0	0
	PDF3	8	0	0
	PDF4	3	0	0
	PDF5	6	0	0
Gaussian filter 3 × 3	PDF1	0	1	0
	PDF2	0	0	0
	PDF3	0	1	1
	PDF4	0	0	0
	PDF5	0	2	0
Scale 2	PDF1	9	0	0
	PDF2	10	0	1
	PDF2	8	0	0
	PDF2	7	0	0
	PDF2	5	0	0

## Data Availability

The study did not report any data.
